# Maternal mosaicism in *SSBP1* causing optic atrophy with retinal degeneration: implications for genetic counseling

**DOI:** 10.1186/s13023-023-02748-9

**Published:** 2023-05-31

**Authors:** Yin-Hsi Chang, Eugene Yu-Chuan Kang, Laura Liu, Laura A. Jenny, Rin Khang, Go Hun Seo, Hane Lee, Kuan-Jen Chen, Wei-Chi Wu, Meng-Chang Hsiao, Nan-Kai Wang

**Affiliations:** 1 Department of Ophthalmology, Chang Gung Memorial Hospital, Linkou Medical Center, Taoyuan, Taiwan; 2 College of Medicine, Chang Gung University, Taoyuan, Taiwan; 3 Graduate Institute of Clinical Medical Sciences, College of Medicine, Chang Gung University, Taoyuan, Taiwan; 4 Department of Ophthalmology, Edward S. Harkness Eye Institute, Columbia University Irving Medical Center, Columbia University, 635 West 165th Street, New York, NY 10032 USA; 5 Division of Medical Genetics, 3Billion Inc., Seoul, South Korea; 6 Department of Pathology and Cell Biology, Columbia University Medical Center, New York, NY USA

**Keywords:** Gonosomal mosaicism, Optic atrophy, SSBP1, Whole exome sequencing

## Abstract

**Background:**

Optic atrophy-13 with retinal and foveal abnormalities (OPA13) (MIM #165510) is a mitochondrial disease in which apparent bilateral optic atrophy is present and sometimes followed by retinal pigmentary changes or photoreceptors degeneration. OPA13 is caused by heterozygous mutation in the *SSBP1* gene, associated with variable mitochondrial dysfunctions.

**Results:**

We have previously reported a 16-year-old Taiwanese male diagnosed with OPA13 and *SSBP1* variant c.320G>A (p.Arg107Gln) was identified by whole exon sequence (WES). This variant was assumed to be de novo since his parents were clinically unaffected. However, WES and Sanger sequencing further revealed the proband’s unaffected mother carrying the same *SSBP1* variant with a 13% variant allele frequency (VAF) in her peripheral blood. That finding strongly indicates the maternal gonosomal mosaicism contributing to OPA13, which has not been reported before.

**Conclusions:**

In summary, we described the first case of OPA13 caused by maternal gonosomal mosaicism in *SSBP1*. Parental mosaicism could be a serious issue in OPA13 diagnosis, and appropriate genetic counseling should be considered.

## Background

Single-strand DNA-binding protein 1 *(SSBP1*, MIM: 600439) gene is a nuclear-encoded housekeeping gene involved in mitochondrial biogenesis [1]. The functional protein binds to single-strand DNA as a tetramer complex to stabilize unwound mitochondrial DNA (mtDNA) and stimulate mtDNA synthesis. Together with mitochondrial polymerase, mtDNA helicase, and mitochondrial RNA polymerase, SSBP1 is responsible for mtDNA replication, repair, and maintenance [2, 3]. Variants in *SSBP1* gene could affect the amount of SSBP1 proteins or disrupt multimer formation [4], and when this happens, mtDNA cannot repair DNA damage properly, leading to mitochondrial dysfunction and diseases.

*SSBP1* variants are associated with a form of inherited optic neuropathies that have phenotypic variabilites manifesting as isolated optic atrophy, optic atrophy combined foveopathy or photoreceptor degeneration [5]. Therefore, it has recently been recognized as optic atrophy -13 with retinal and foveal abnormalities (OPA13) (MIM #165510). The *SSBP1* gene was functionally characterized in 2019 and several families with OPA13 have been reported recently [4, 6, 7]. *SSBP1* mutations could impair replication machinery in retinal ganglion cells (RGCs) and numerous other cell types [8]. Although OPA13 has been recognized as an autosomal dominant disorder [4–7, 9], the *SSBP1* genotype–phenotype correlations are not well understood.

In our previous work, a 16-year-old Taiwanese male with bilateral disc pallor, retinal vessel attenuation carried an *SSBP1* variantc.320G>A (p.Arg107Gln) identified by whole exome sequence (WES) [8]. Here, we further discovered that the proband’s disease-causing variant was inherited from his unaffected mother, strongly indicating the maternal gonosomal mosaicism contributing to OPA13.

## Methods

Clinical data of the proband was obtained and evaluated at Chang Gung Memorial Hospital Medical Center from 2010 to 2022. His parents underwent comprehensive ophthalmic examinations including best-corrected visual acuity (BCVA), intraocular pressure, slit lamp, fundus examination, color fundus photography, and spectral domain optical coherence tomography (SD-OCT) (Heidelberg Engineering, Heidelberg, Germany). DNA was extracted from peripheral blood using the QIAamp DNA Mini Kit (Qiagen Inc., Valencia, CA). Samples were analyzed using Sanger sequencing and WES. The study was conducted in accordance with the tenets of the Declaration of Helsinki and approved by the Institutional Review Board of Chang Gung Memorial Hospital (No. 201601569B0C602).

## Results

**Fig. 1:**
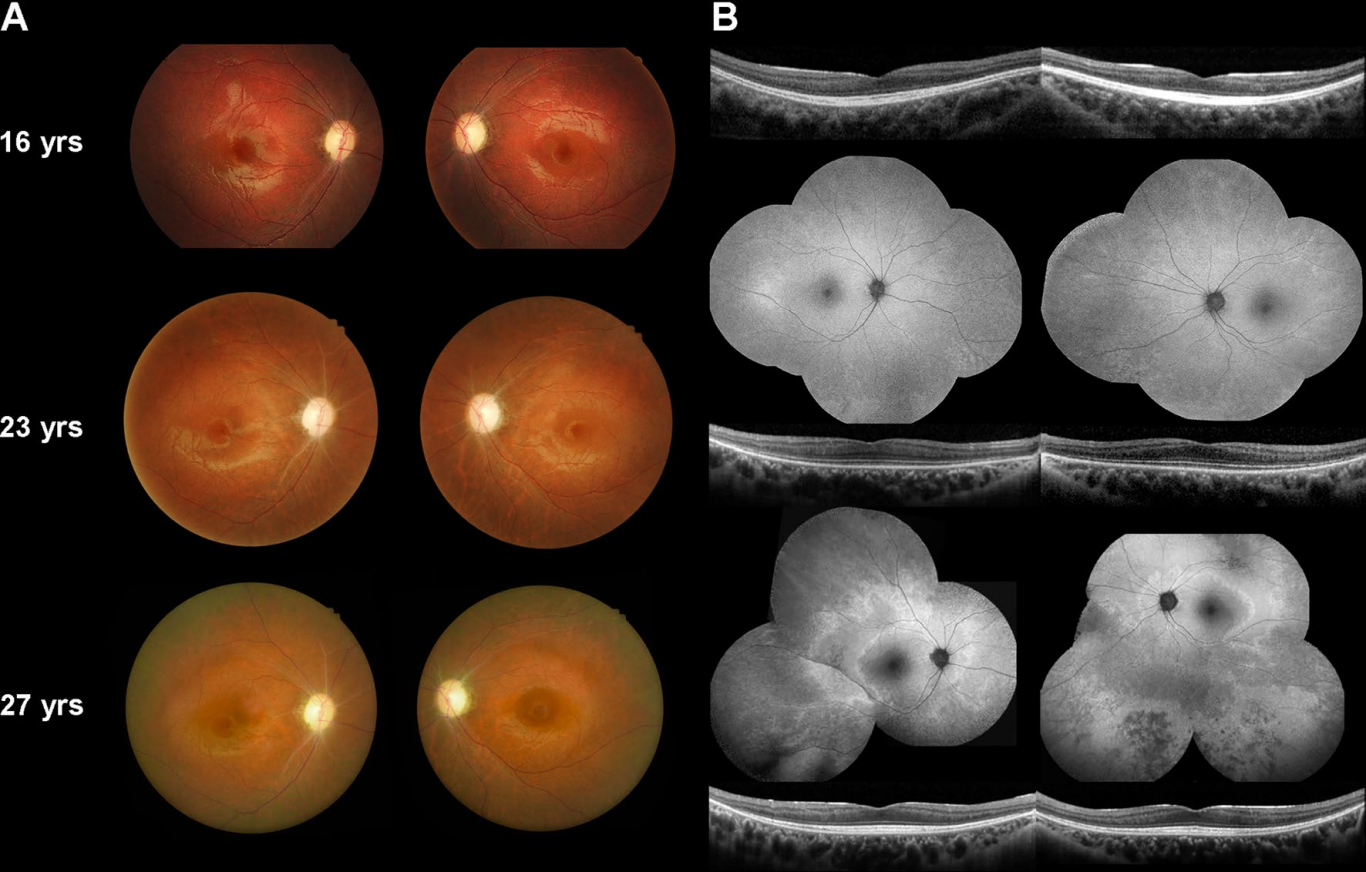
Retinal images. **A** Color fundus photography of our patient with OPA13 at 16, 23 and 27 years old. **B** Spectral domain-optical coherence tomography of the macula at 16, 23 and 27 years old, and autofluorescence imaging at 16 and 27 years old

A 16-year-old Taiwanese male presented to our department with poor vision, fair night vision, and color blindness in both eyes since childhood. The BCVA was 20/100 in the right eye and 20/200 in the left eye; intraocular pressure and anterior segment was normal in both eyes. Dilated fundus examination showed bilateral optic disc pallor and retinal vessel attenuation without obvious pigmentation at the initial presentation (Fig. 1A). Autofluorescence imaging was non-contributory, and SD-OCT of the macula revealed no structural abnormality except RNFL thinning (Fig. 1B). Electrophysiological tests showed early optic nerve dysfunction followed by gradual loss of photoreceptor response as described in our previous study [8]. When the patient repeated exams by age 27, mid-peripheral retinal pigmentary changes appeared on fundus color photography and autofluorescence imaging (Fig. 1B), which supported the hypothesis that RGCs are affected earlier than photoreceptors. The outer retina layers did not show disruption but relative hypo-reflection was noted at the cone outer segment tips (COST) line on SD-OCT of the macula (Fig. 1B).

WES detected a pathogenic heterozygous *SSBP1* missense variant (NM_003143.3) c.320G>A (p.Arg107Gln), confirming the diagnosis of OPA13 with retinal and foveal abnormalities (MIM #165510). This variant has been reported in several OPA13 individuals with an autosomal dominant inheritance pattern and has been classified as pathogenic in ClinVar (Variation ID: 977503) [6, 7, 10].

The patient reported no family history of visual disorders, night blindness or consanguinity. The patient and his parents were otherwise healthy and had no other ocular or systemic diseases. His 51-year-old mother had a VA of 20/22 in the right eye and 20/40 in the left eye. His 56-year-old father presented with 20/28 in the right eye and 20/66 in the left eye. Color fundus photography and SD-OCT imaging were normal. Therefore, it was expected that the proband’s variant was de novo*.*

**Fig. 2:**
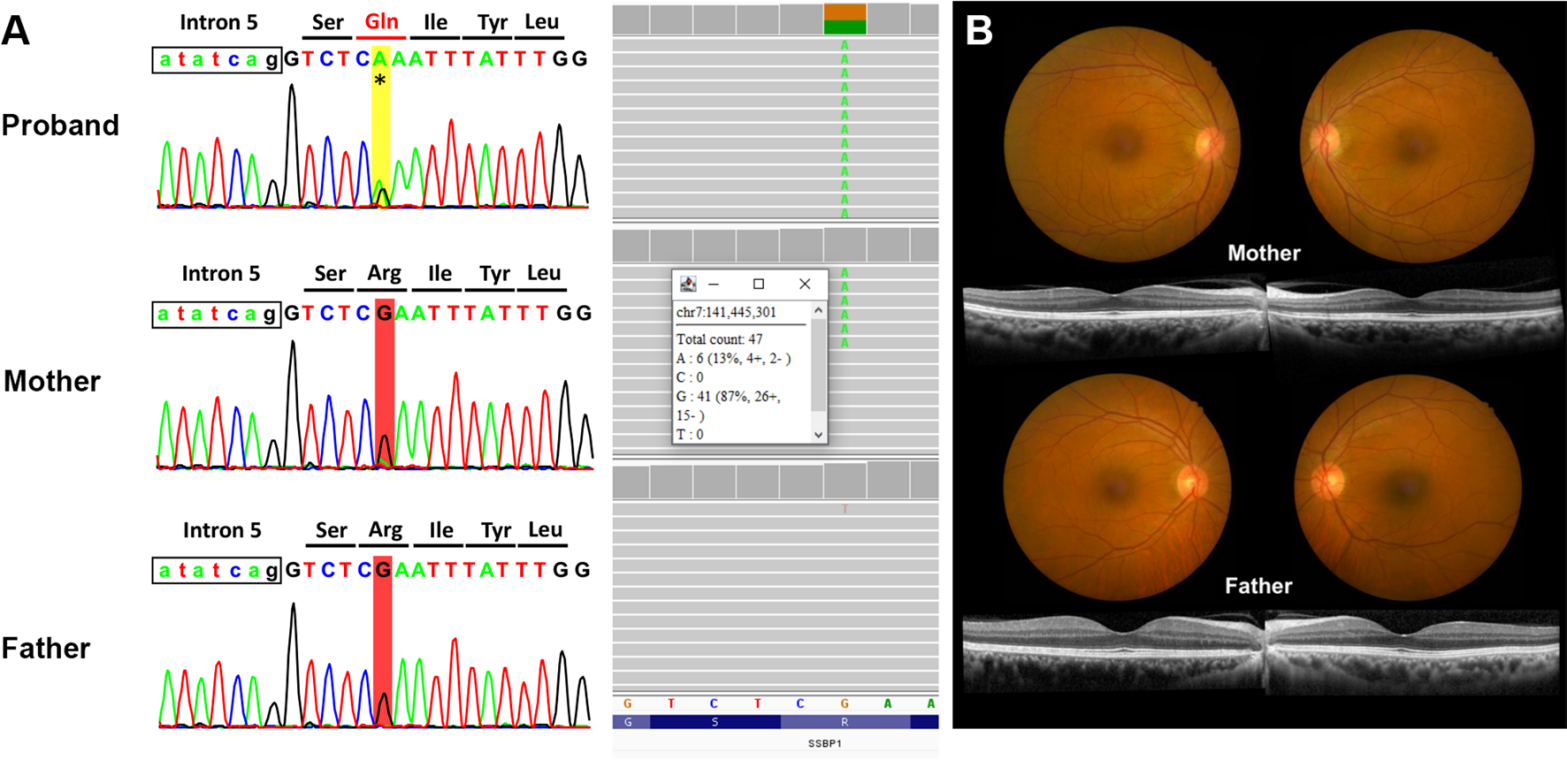
Family studies. **A** Sanger sequencing (left) and whole exome sequencing (right) of the patient and his parents. *SSBP1* variant c.320G>A (p.Arg107Gln) was identified in the proband (first row), but equivocal in his mother (second row) using Sanger sequencing. Whole exome sequencing revealed a 13% of variant allele frequency of the patient’s mother. His father’s result is negative (third row). **B** Color fundus photography and spectral domain-optical coherence tomography of the macula of the parents are normal

However, by Sanger sequencing analysis, an unusual trace of a single nucleotide A was observed at the variant position in the proband’s unaffected mother. This trace was stronger than what is usually considered sequencing noises at other nearby positions. This finding was confirmed by trio WES analysis, showing that the mother also carried the same *SSBP1* variant with a ~ 13% variant allele frequency (VAF) in her peripheral blood, and the father carried wild-type alleles at this position. The trio WES analysis did not find any additional clinically significant variants. We repeated trio Sanger sequencing, and the results were consistent with WES, indicating a maternal mosaicism of the variant. This low-level mosaic variant could be detected with careful evaluation of both WES and Sanger sequencing (Fig. 2A).

## Discussion

Mutational events that occur during early embryonic mitosis can cause both somatic and germline mosaicism, also known as the gonosomal mosaicism [11]. Here, we described a maternal gonosomal mosaicism in *SSBP1* as the disease-causing mechanism of OPA13. Initially it was mis-assumed that the variant would be de novo because of a negative family history. De novo variants (DNVs) refer to germline or somatic variants discovered in the offspring that cannot be detected in the genome of either parent [12]. Interestingly, about 3.54% (range 0.22 to 6.15%) of presumed germline DNVs originate from parental mosaicism [13]. Differentiating the origination of the variant is important to help predict the recurrence risk in the family. The average recurrence probability of pathogenic DNVs in an additional child is typically estimated to be 1.3%. However, it increases to 24% for DNVs that are mosaic in > 1% of parental blood cells and 50% for DNVs mosaic in > 6% of parental blood cells [14]. The discovery of a low-level mosaic variant in the unaffected mother suggests that the variant was inherited and therefore the risk of occurrence in future offspring increases. Including information about gonosomal mosaicism is crucial for providing accurate genetic counseling to families with SSBP1 variants. Family members are able to gain more comprehensive information about the nature and inherited pattern of the pathogenic variants. Genetic counseling can help the family understand the risk of occurrence and make informed decisions about their reproductive options or prenatal testing.

Although parental mosaicism has been reported in different genetic disorders, parental mosaicism in *SSBP1* has not been reported before. The term mosaicism refers to the presence of two or more genetically different cell populations within one individual as a result of post-conceptual mutation [15]. Depending on the timing of the post-zygotic mutation, the distribution of mutant cells in the individual is different. In this study, the unaffected mother carried a low-level of mosaic *SSBP1* variant in her blood, and the same variant was identified in her affected son, indicating a maternal gonosomal mosaicism contributing to OPA13. Recent studies have found that transmission of parental postzygotic mosaicism could explain up to 10% of DNVs in rare neurodevelopmental diseases, which is more frequent than previously predicted [16, 17]. Low-level variants present in an individual's cells can be challenging to detect by standard diagnostic techniques [18]. Low-level mosaicism may not be detected by Sanger sequencing because the lower limit of detection is generally recognized as being approximately 15% to 20% VAF [19–21]. In order to provide a more accurate diagnosis, additional testing such as WES or digital polymerase chain reaction (PCR) is recommended to confirm low-level mosaic variants.

Wide variability in phenotypes has been noticed in OPA13, and the *SSBP1* genotype–phenotype correlations is not well-established yet. Among 60 published patients with missense *SSBP1* variants, optic atrophy was present in 95% of them [4–8]. Sixty-five percent of patients had pigmentary changes on fundus photography, 57.8% presented with foveopathy on macular OCT, and 29.3% exhibited rod-cone degeneration on full-field ERG. There are two possible mechanisms that may contribute to this variability. First, somatic or gonosomal mosaicism in the patient may explain part of the phenotypic variability or reduced penetrance, as described in other dominant ocular disorders [22]. The type and percentage of retinal cells involved could differ. The clinical outcome is assumed to be related to the mosaic mutant allelic fractions [23]. Second, the dominant-negative effect of the mutation can result in variable phenotypes as well. Jurkute et al. suggested that mutant SSBP1 functions as a dominant-negative protein interfering with the assembly of functional multimers [7, 24]. Thus, the variability of clinical presentations could be associated with the expression levels of the mutant alleles versus the trans alleles. Given that dominant-negative mutations usually cause severer effects than those of simple null alleles, other genetic mechanisms such as haploinsufficiency cannot be excluded. Future experiments using animal models are needed to elucidate the mechanisms underlying *SSBP1* mutations.

## Conclusion

In summary, our study revealed the first case of OPA13 caused by maternal gonosomal mosaicism in *SSBP1*. Additionally, we described comprehensive ocular examinations with 11-year follow-up in a young male diagnosed with *SSBP1*-related OPA13 of what was originally believed to be a de novo mutation in a patient with OPA13. This study highlights the importance of accurately detecting parental somatic mosaicism followed by appropriate genetic counseling, as low-level mosaicism may result in misinterpretation of the risk of recurrence. It is particularly challenging for diagnostic laboratory testing to detect low-level mosaic variants due to the limits of detection sensitivity and additional testing may be required.

## Data Availability

The dataset used and/or analysed during the current study is available from the corresponding author on reasonable request.
